# QUALITY OF LIFE IN PATIENTS UNDERGOING REVERSE SHOULDER ARTHROPLASTY

**DOI:** 10.1590/1413-785220253304e290152

**Published:** 2025-09-08

**Authors:** Gabriela Rezende Spini, Fernando Pascolat Castro, Gabriel Willian Berger Melo, Pedro Ivo Santos Aranas

**Affiliations:** 1 Nova Bene - Beneficencia Portuguesa de Ribeirao Preto, Sao Paulo, SP, Brazil.

**Keywords:** Arthroplasty, Replacement, Shoulder, Shoulder Fractures, Shoulder Injuries, Quality of Life, Artroplastia do Ombro, Fraturas do Ombro, Lesões do Ombro, Qualidade de Vida

## Abstract

**Objective:**

To identify and review national and international articles that address the impacts on quality of life and the main clinical-functional outcomes of patients undergoing rotator cuff arthroplasty technique.

**Methods:**

A systematic literature search was conducted using the databases Lilacs, MedLine, Pubmed, Scielo, BVS and Cochrane, published in the last 05 years.

**Results:**

623 articles were found, with 575 excluded after temporal screening, title and abstract reading. For initial registration purposes, 48 studies were selected, of which 12 were excluded for being duplicates. Of the remaining 36 studies, 31 were excluded for not meeting the inclusion criteria, resulting in 05 studies that composed the synthesized data. All classified as Evidence Level I by AHRQ.

**Conclusions:**

The results of this study suggest that individuals with rotator cuff injuries can benefit from the Reverse Shoulder Arthroplasty technique for rotator cuff treatment, where it can be observed that patients undergoing RSA had significant improvement in functional capacity and quality of life, showing improvements in both physical and emotional aspects and functional independence. ***Level of Evidence III; Systematic Review***.

## RESUMO

**Objetivo:**

Identificar e revisar artigos nacionais e internacionais que abordam os impactos na qualidade de vida e os principais resultados clínico-funcionais de pacientes submetidos à técnica de artroplastia do manguito rotador.

**Métodos:**

Foi realizada busca sistemática de literatura, a partir das bases de dados Lilacs, MedLine, Pubmed, Scielo, BVS e Cochrane, publicados nos últimos 05 anos.

**Resultados:**

Foram encontrados 623 artigos, sendo 575 excluídos após a realização de recorte temporal, leitura de títulos e resumo. Para fins de registro inicial foram selecionados 48 estudos, dos quais 12 destes foram excluídos por estarem em duplicidade. Dos 36 estudos restantes, 31 foram excluídos por não atenderem aos critérios de inclusão, resultando em 05 estudos que compuseram os dados sintetizados. Todos classificados com Nível de Evidência I pela AHRQ.

**Conclusões:**

Os resultados deste estudo sugerem que indivíduos com lesões do manguito rotador podem se beneficiar da técnica da Artroplastia Reversa do Ombro, para o tratamento do manguito rotador, onde pode-se observar que pacientes submetidos à ARO tiveram melhoria significativa na capacidade funcional e qualidade de vida, apresentando melhorias tanto no aspecto físico, quanto emocional e de independência funcional. ***Nível de Evidência III; Revisão Sistemática***.

## INTRODUCTION

Rotator cuff arthroplasty represents a spectrum of shoulder diseases characterized by rotator cuff insufficiency, decreased distance from the humeral head to the acromion, subacromial impingement and arthritic changes in the glenohumeral joint. The initial treatment should be conservative and the possibilities of intervention, when necessary, range from arthroscopic debridement, hemiarthroplasty, reverse arthroplasty and arthrodesis or resection arthroplasty, both in extreme cases.^1,2^

It has widely documented benefits in degenerative pathologies of the glenohumeral joint, since biomechanically it improves the functioning of the deltoid muscle, moving it distally in order to provide a greater lever arm with an increase in its perpendicular distance to the center of joint rotation, which due to the shape of the semi-constrictor remains stable and compensates for the dysfunctional rotator cuff, Reverse shoulder arthroplasty initially emerged as an alternative technique for various shoulder conditions/injuries, becoming an option for patients with proximal humeral fractures, rheumatoid arthritis, fixed glenohumeral dislocation, tumor surgery, fracture pseudarthrosis, glenoid bone loss and/or revision arthroplasty.^2-6^

Affecting mainly women over 60 years of age, and initially intended to treat shoulder osteoarthritis with rotator cuff deficiency in elderly patients with loss of active lifting of the arm (pseudo-paralytic shoulder), this type of procedure has revolutionized the reconstructive surgery of this joint and, due to promising clinical results, has become increasingly common in the treatment of arthritic conditions, so that studies point out that in recent decades, the rotator cuff arthroplasty see presenting superior results compared to hemiartroplasty.^2,7-10^

Noting promising results in the scenario of proximal fractures of the humerus leading to fewer restrictions during the immediate postoperative period, considering that the lack of healing of tuberosity does not lead to functional disaster as seen in hemiarthroplasty and even if it requires more time and intraoperative effort on the humeral side on the glenoid can be easily exposed to allow the proper placement of the base plate and the glenosphere, resulting in better functional results.^3,11-13^

On the other hand, it is considered as the perception of the individual of his position in life in the context of the culture and system of values in which he lives and in relation to his goals, expectations, patterns and concerns and even as an ethical question, which must, primarily, be analyzed from the individual perception of each. The concept of Quality of Life (QL) has gained increasing importance in the field of healthcare since the mid-1980s, having a significant increase in medical discourse and practice.^14-17^

It is defined by the World Health Organization (WHO) as "the individual's perception of their insertion in life, in the context of the culture and values systems in which they live and in relation to their goals, expectations, patterns and concerns". QL therefore involves both spiritual, physical, mental, psychological and emotional well-being, as well as social relationships, such as family and friends and also health, education, basic sanitation housing and other life circumstances.^18^

Essential for the recovery of patients undergoing reverse shoulder arthroplasty, QL is closely linked to well-being and success in the treatment of these patients. In this sense, it becomes relevant, through this systematic review study, to identify and review national and international articles that address the impacts on their quality of life and the main clinical-functional results of patients undergoing the arthroplasty technique of the rotator cuff.

## MATERIAL AND METHOD

This review was conducted according to the Preferred Reporting Items for Systematic Reviews and MetaAnalyses (PRISMA) methodology.^19^

According to the Oxford Centre for Evidence-Based Medicine, systematic reviews of randomized and controlled clinical trials are considered the best level of scientific evidence (A1) when therapies are evaluated.^20^ This is due to the fact that randomized and controlled trials are considered with excellent level of evidence and the systematic review is a compilation of data obtained from several of these papers on the same subject.^21^

For the elaboration of this review, the following steps were considered: development of the research question; search in the databases; selection of the articles; extraction of data; evaluation of the methodological quality; synthesis of the data, evaluation of the quality of the evidence; drafting and publication of the results.

Moreover, respecting what was proposed to evaluate, the guiding question was: to evaluate the main clinical-functional results of patients undergoing the technique of reverse shoulder arthroplasty, in addition to the impacts on their quality of life of these patients after surgical procedure.

### Search strategy

We conducted a systematic search of literature from Latin American and Caribbean Literature in Health Sciences (Lilacs), Medical Literature Analysis and Retrieval System Online (MedLine/Pubmed), Scientific Electronic Library Online (Scielo), Virtual Library in Health (BVS) and Cochrane Library.

As a timescale, the research was based on the analysis of studies published over the past five years.

The descriptors used in the search strategy were identified based on PubMed and replicated to the other libraries and databases, using the following combinations of terms: Arthroplasty (*Arthroplasty*); Shoulder Arthroplasty (*Arthroplasty, Replacement, Shoulder*); Shoulder Fractures (*Shoulder Fractures*); Shoulder Injuries (*Shoulder Injuries*) and Quality of Life (*Quality of Life*).

All descriptors and their synonyms have been combined with each other. For the descriptors, the combinations were made using the Boolean term "AND", while for the synonyms, the Boolean term "OR" was used.

It should be noted that the above descriptors are found in the Descriptors in Health Sciences (DeCS).

### Eligibility criteria

As eligibility criteria, studies available in Portuguese, English or Spanish that answered the guiding question and published in the last five years were included.

Studies conducted on animals, studies of narrative review, magazines, newspapers and/or books that did not meet the proposed study were excluded.

### Selection of articles

The articles were downloaded through the Chrome browser. The files were selected individually by two distinct authors. Disagreements in the selection of articles were resolved by mutual agreement.

The research of the articles took place during the first quarter of 2024. The following variables were included in the data extraction tool: Title/Theme; Author(s); Year/ Country; Objectives; Study Design/ Evidence Level; Results and Conclusion.

These were then presented by means of tables and/or tables contemplating the main characteristics of the articles used for the purposes of this review.

## RESULTS

623 articles were found, of which 575 were excluded after timing, reading titles and summary.

For initial registration purposes, 48 studies were selected for analysis, of which 12 were excluded because they were in duplicity.

**Figure 1:**
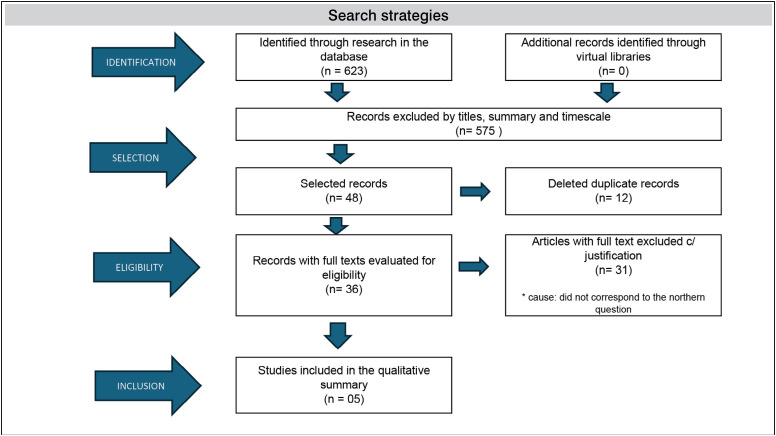
Flowchart of the study selection process.

Source: Authors.

Of the remaining 36 studies, 31 were excluded because they did not meet the inclusion criteria, resulting in 05 (five) studies that compiled the synthesized data (Figure 1).

For eligibility purposes and proposed results, studies of systematic review, metanalysis, randomized clinical study, cohort study, clinical trial and observational study were initially listed. Only the systematic review and metanalysis studies are retained, so that they are part of Evidence Level I, as established by the Agency for Healthcare Research and Quality (AHRQ).

The critical analysis of the studies conducted using the categorization by evidence levels by the AHRQ, covers seven levels: (I) evidence from metanalysis and systematic review; (II) evidence from clinical trials with randomization; (III) evidence from clinical trials without randomization; (IV) evidence from cohort and case-control studies; (V) evidence from systematic review of descriptive and qualitative studies; (VI) evidence based on descriptive or qualitative study and (VII) opinions from authority or expert committee.^22^

Among the selected articles, the oldest was published in 2019 and the most recent in 2024. Of the total articles included, (n=05, 100%) were available in English. As for the origin, articles with full text prevailed (n = 04, 100%).

Finally, with respect to the study design/evidence level according to the AHRQ categorization, (n=05, 100%) of these corresponded to the level of evidence Level I (Table 1).^23-27^

**Table 1:** Summary of the publications used in this review.

No	Title	Author(s)	Year	Country	Language	Study Design / Evidence Level
1	The clinical impact of retears after repair of posterosuperior rotator cuff tears: a systematic review and meta-analysis	Holtedahl et al.^23^	2023	United States	English	Systematic review and metanalysis Level I
2	The Relationship of Aging and Smoking With Rotator Cuff Disease: A Systematic Review and Meta-analysis	Grusky et al.^24^	2022	United States	English	Systematic review and metanalysis Level I
3	Conservative versus surgical management for patients with rotator cuff tears: a systematic review and META-analysis	Longo et al.^25^	2021	United States	English	Systematic review and metanalysis Level I
4	Shoulder replacement surgery for osteoarthritis and rotator cuff tear arthropathy	Craig et al.^26^	2020	United States	English	Systematic review and metanalysis Level I
5	Surgery for rotator cuff tears	Karjalainen et al.^27^	2019	United States	English	Systematic review and metanalysis Level I

## DISCUSSION

Mostly present in elderly patients over 60 years, studies by Grusky et al.^24^ aimed at synthesizing evidence from studies that report associations between aging and smoking in relation to rotator cuff disease, point out that the increase in age is considered a strong risk factor for rotator cuff disease and that current smokers are more likely to have rotator cuff disease compared to non-smokers and/or ex-smokers.

However, the results point out that despite age, and that the initial treatment recommendation for this patient profile should always be conservative, with changes in activities, oral analgesics, physiotherapy and/or intraarticular infiltrations. Usually, surgical treatment becomes necessary in patients with shoulder lesions. Since ARO has proved to be an excellent option for the treatment of patients with arthroplasty of the rotator cuff with satisfactory functional results.^28^

Developed primarily for the treatment of rotator cuff arthroplasty, reverse shoulder arthroplasty comprises in the surgical technique for the treatment of various shoulder conditions/lesions through the replacement of the damaged cartilage surface, creating new slippery and painless surfaces, thereby aiming to relieve pain and improve shoulder movement.^3^ And this is possible through the proper balance of soft parts, the correct choice of the implant and the restoration of the articular anatomical parameters.^29^

Karjalainen et al.^27^ in systematic review studies aimed at synthesizing the available evidence on the benefits and disadvantages of repairing the rotator cuff with or without subacromial decompression in the treatment of shoulder rotator cuff fractures, point out that the overall success rate evaluated by the participants was 873/1,000 after non-operative treatment and 943/1,000 after surgery, corresponding to (risk rate (RRR) 1.08, confidence interval (IC) from 95% 0.96 to 1.22; evidence of low quality (reduced by bias and inaccuracies). And that health-related quality of life was 57.5 points (SF-36 mental component score 0 to 100, higher score indicating better quality of life) with non-operative treatment and 1.3 points worse (4.5 worse to 1.9 better) with non-operative treatment surgery (1 study; 103 participants), low-quality evidence (reduced by bias and inaccuracies). No, and it is therefore possible to estimate the risk of adverse events and serious adverse events, as only one event was recorded in the trials (very low-quality evidence; reduced once due to biases and twice due to very serious inaccuracies).

For France et al.^30^ although ARO is a relatively new procedure in Brazil, it is a procedure that can be used effectively and safely in patients who previously presented themselves without therapeutic options such as arthroplasty of the rotator cuff and revisions that provide pain relief, improved function and upper limb mobility.

Corroborating with studies by Amaral et al.^31^ which state that ARO consists of the procedure that restores shoulder joint function in patients who previously presented themselves without therapeutic options. And whose technique aims to provide better quality of life and mobility for patients.

For Craig et al.^26^ in systematic review studies of metanalysis, aiming at determining the benefits and disadvantages of shoulder replacement surgery in adults with shoulder osteoarthritis (OA), including rotator cuff rupture arthroplasty (RCTA), according to authors, although shoulder total replacement surgery is an established procedure, no high-quality randomized trial has been conducted to determine whether shoulder replacement can be more effective than other treatments for osteoarthritis or shoulder rotator cuff rupture arthroplasty. It remains unclear, therefore, which type and/or technique of shoulder replacement surgery is most effective in different situations.

Corroborating with studies by Longo et al.^25^ which, when comparing conservative versus surgical management for patients with complete rupture of CR in terms of clinical and structural results in 1 and 2 years of follow-up, report that in the follow-up of 2 years, the shoulder function evaluated in terms of CMS did not improve significantly. Where the mean value of the CMS score at 12 months follow-up was 77.6 ± 14.4 in the surgical group and 72.8 ± 16.5 in the conservative group, i.e., without statistically significant differences between the groups. High-quality, randomized, level I clinical trials with long-term follow-up are recommended to assess whether surgical and conservative treatment really provides comparable results in the long term.

Holtedahl et al.^23^ in a systematic review of metanalysis aimed at analyzing the relations between postoperative rotator cuff integrity and shoulder pain and function, even stated that the negative impact of relapses on pain and function was statistically significant, but considered of less clinical importance. As such, the results indicate that most patients can expect satisfactory results despite the new ruptures.

In addition, Kim et al.^32^ in studies aimed at analyzing the clinical results of reverse total shoulder arthroplasty (RTSA), according to the primary diagnosis, highlight that based on excellent RTSA results in patients with arthroplasty for rotator cuff tear, the indications for this treatment method were broadened as implants are improved and surgeons gain more experience, and RTSA has been used for treatment and review of other diseases and fractures.

Corroborating with Maia et al.^33^ which state that considering that the main indication for reverse arthroplasty is for the patient with rotator cuff arthroplasty presenting pain and loss of shoulder movement arc and due to the good results obtained in the treatment of this pathology, the indications for the use of reverse arthroplasty have gradually expanded to include other conditions that were previously difficult to treat successfully and predictably.

On the other hand, being approached by many authors, as a synonym for health, and by others as a more comprehensive concept, in which health conditions would be one of the aspects to be considered.^34,35^ The QV, is therefore a multidimensional concept, which includes economic issues, lifestyle, health conditions, housing, personal satisfaction and social environment, among others, and is often used as a synonym for health.^36,37^

And in patients undergoing reverse shoulder arthroplasty (ARO), according to studies by Leite et al.^38^ with 35 patients undergoing ARO, the results showed good quality of life related to the health of these patients, considering both the mental and physical scores they were undergoing.

Studies by Ribeiro et al.^39^ involving 28 patients in clinical evaluation after undergoing ARO, indicate that reverse shoulder arthroplasty presents, in the short and medium term, satisfactory functional results, which are influenced by sex, age to the procedure, follow-up time and pathology that led to the indication.

Corroborating with studies from Figueira et al.^40^ that in a systematic review of metanalysis aimed at evaluating the results of ARO in QV of elderly patients, the results highlighted that functional capacity, ability to fully carry out their daily life activities, autonomy and functional independence is closely linked to QV of these patients.

## FINAL CONSIDERATIONS

The results of this study suggest that individuals with rotator cuff lesions can benefit from the reverse shoulder arthroplasty technique for the treatment of the rotator cuff, where it can be observed that patients undergoing ARO had significant improvements in functional capacity and quality of life, showing improvements in both physical, emotional and functional independence.
